# Effect of various dentin disinfection protocols on the bond strength of resin modified glass ionomer restorative material

**DOI:** 10.4317/jced.53725

**Published:** 2017-07-01

**Authors:** Anand Sekhar, Akansha Anil, Manuel S. Thomas, Kishore Ginjupalli

**Affiliations:** 1 Post graduate student, Dept. of Oral and Maxillofacial Surgery, Yenepoya Dental College, Yenepoya University, Deralakatte, Mangalore, Karnataka. Former student of Manipal College of Dental Sciences, Manipal University, Mangalore, Karnataka; 2 Post graduate student, Dept. of Periodontics, College of Dental sciences, Davangere, Karnataka. Former student of Manipal College of Dental Sciences, Manipal University, Mangalore, Karnataka; 3 Associate Professor, MDS, Dept. of Conservative Dentistry and Endododntics, Manipal College of Dental Sciences, Manipal University, Mangalore; 4 MSc, PhD, Dept. of Dental Materials, Manipal College of Dental Sciences, Manipal University, Manipal

## Abstract

**Background:**

Disinfection of dentin surface prior to any restorative therapy is important for the longevity of the treatment rendered. However, these dentin disinfection methods should itself not interfere with the adhesion of the restorative material. Therefore the aim of this study was to determine the effect of various dentin disinfection protocols on the shear bond strength (SBS) of resin modified glass ionomer cement (RMGIC).

**Material and Methods:**

The occlusal surface of 40 extracted premolars were trimmed to obtain a flat dentinal surface and was randomly divided into four groups. CTRL was the control group; NaOCl was 1% sodium hypochlorite disinfection group; CHX was 2% chlorhexidine disinfection group; and PAD was the photo-activated disinfection group. Then a predetermined dimension of RMGIC was bonded to the pre-treated dentin surfaces. Following this, each sample was tested for SBS using universal testing machine at a crosshead speed of 0.5mm/min.

**Results:**

Among the test groups, CHX showed the least reduction in SBS and NaOCl the highest reduction in SBS as compared to the control group. PAD on the other hand showed significantly lower SBS than CTRL and CHX groups, but the values were higher than the NaOCl group.

**Conclusions:**

Thus, it could be concluded from the present study that use of chlorhexidine based dentin disinfection does interfere with the adhesion of RMGIC. However, photo-activated disinfection should be done with caution. Moreover, sodium hypochlorite based disinfectants should be avoided prior to the use of RMGIC.

** Key words:**Chlorhexidine, Dentin disinfection, Photo-activated disinfection, Resin modified glass ionomer cement, Shear bond strength, Sodium hypochlorite.

## Introduction

Wilson and Kent introduced glass ionomer cement (GIC) to the field of dentistry. Over the years, the original composition of GIC has been modified to achieve superior initial mechanical strength by incorporating polymerizable resins. This light cure form of GIC is widely known as resin modified glass ionomer cement (RMGIC). It has desirable properties similar to conventional GIC like biocompatibility, fluoride release, anti- microbial activity, co-efficient of expansion similar to that of tooth and physio-chemical bond with the tooth structure. Apart from this, RMGIC exhibits command set, superior early mechanical strength and reduced sensitivity to moisture (1). These qualities make RMGIC an excellent restorative material in low-stress bearing areas, especially for patients who are at a higher risk for caries.

The purpose of tooth preparation in restorative dentistry, in addition to providing adequate space for the restorative material, is to remove the infected dentin. Residual bacteria remaining within the cavity after caries excavation can cause recurrent caries and can affect the pulp (2,3). Additionally in endodontics, adequate sterilization of the pulp space in mandatory to prevent failure of the treatment (4). The problems associated with incomplete disinfection of the prepared cavity or the pulp space can be exaggerated if microleakage is present at the tooth-restoration interface. Therefore the factors that adversely affect the bonding of restorative materials can increase the risk for microleakage, and this in turn can negatively impact the longevity of treatment rendered. For that reason complete elimination of microorganisms present within the prepared cavity without any interference with the adhesion of restorative material is essential for the predictable prognosis of treatment (5) .

A potential method by which the residual microorganisms can be eliminated is with the use of antibacterial agents within the prepared cavity just before the placement of restorative material. Various chemicals that have been tested as cavity disinfects, include chlorhexidine digluconate, sodium hypochlorite, hydrogen peroxide, iodine potassium iodide, etc (5,6). A newer and effective way to eliminate the microorganisms from within the dentin is the use of photo- activated disinfection, where an appropriate wavelength of light is used in conjunction with a photo-sensitizer to produce singlet oxygen and other radical species to cause rapid and selective destruction of the target cells (7-9). However it is important that these cavity disinfection methods do not interfere with the adhesion of the restorative materials used.

Therefore the aim of this in-vitro study was to assess the effect of various dentin disinfection protocols on the shear bond strength of RMGIC.

## Material and Methods

-Sample selection and storage: Forty non-carious extracted human premolars with no wear defects, fracture line, or cracks were included for the study. Soft tissues, if any attached to the selected teeth were removed using a hand scaler and stored in distilled water until use.

-Sample preparation: The teeth were embedded on to self-cure acrylic resin with only the crown portion visible. A flat dentinal surface parallel to the occlusal plane was obtained using a diamond cutting disc attached to a slow speed micro motor hand-piece. The tooth surface was made even using 180-grit silicon carbide abrasive paper and then polished with a 600-grit silicon carbide paper to standardize the smear layer.

**Figure 1:**
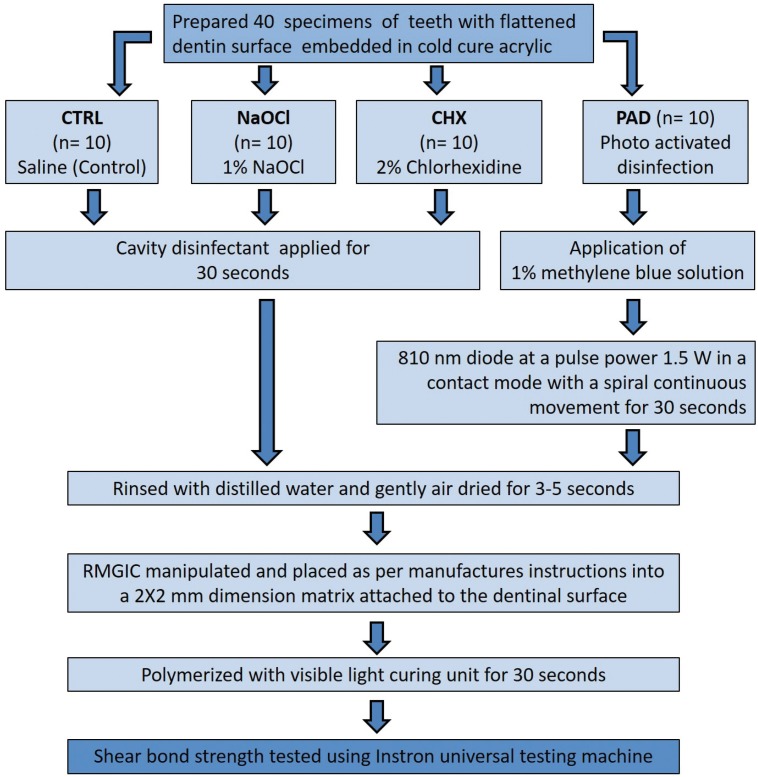
Flow chart representing the experimental procedure and test groups.

-Groups: The samples were assigned randomly to four treatment groups with 10 teeth per group (Fig. 1).

1. CTRL: In the control group no disinfection protocol was followed. The dentinal surface of the samples was rinsed with distilled water and gently air dried for 5 seconds.

2. NaOCl: The dentinal surface were treated with 1% sodium hypochlorite solution (Novo Dental Products Pvt. Ltd., Mumbai, India) for 30 seconds. The surface was then rinsed with distilled water and gently air dried for 5 seconds.

3. CHX: The dentin surfaces were treated with 2% chlorhexidine digluconate solution (Asep-RC, Anabond Stedman Pharma Research (P) Ltd, India) for 30 seconds. The surface was then rinsed with distilled water and air dried for 5 seconds.

4. PAD: 1% methylene blue solution (Qualigens Fine Chemicals, Mumbai, India) was applied on to the dentin surface. The surface was then treated with diode laser (ADV Laser, Picasso, Italy) that provided a monochromatic light of 810 nm at a power setting of 1.5 W in a continuous mode. The laser was delivered through a flexible optic fiber tip of 400µm which was held perpendicular to the dentin surface. This was used in a light contact mode with a continuous spiral movement for 30 seconds with a 5 second break in between for each sample. The dentin surface was then rinsed with distilled water and gently air dried for 5 seconds.

-Placement of RMGIC: The surface was then conditioned with 10% polyacrylic acid (GC Corporation, Tokyo, Japan) for 10 seconds. The dentin surface was again rinsed and dried. Resin modified glass ionomer cement (Fuji II LC, GC Corporation, Tokyo, Japan) was manipulated as per the manufacturer guidelines. It was packed into a cylindrical shaped plastic matrix of 2 mm height and 2 mm internal diameter attached to the center of the treated dentinal surface. The specimens were then cured for 30 seconds using Elipar 2500 light curing unit (3M ESPE, St. Paul, MN). The samples were stored for 24 hours at a temperature of 37ºC and 100% humidity before the bond strength measurements.

-Shear bond strength analysis: The samples were then placed into a positioning jig and tested in shear with an Universal Testing Machine (3366, Instron Corporation, Canton, MA) at a crosshead speed of 0.5 mm/min. Maximum load to debond the RMGIC from the dentin for each specimen was noted and the same was divided by the bonding area to obtain the bond strength in MPa.

-Statistical analysis: The mean and standard deviation of the shear bond strength (SBS) in each group were calculated. Inter group comparison was done using One –way ANOVA test and multiple comparisons were performed using Tukey HSD using SPSS version 17 (SPSS Inc., Chicago, IL, USA). The level of significance was set at *P* <0.05 value.

## Results

**Figure 2:**
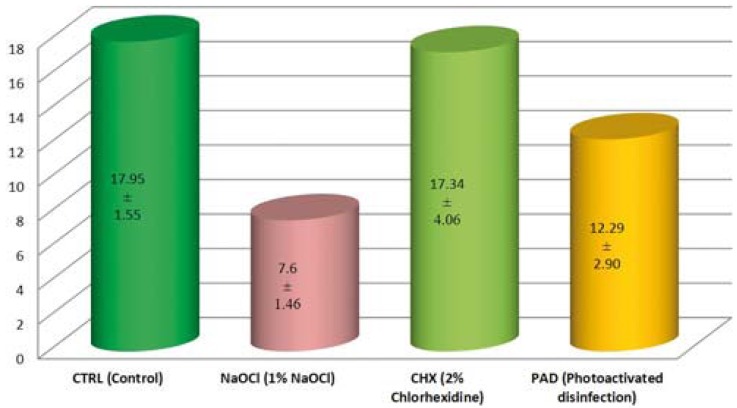
Mean bond strength (in MPa) of RMGIC in various groups.

Among all the groups, the control group showed the highest SBS (17.95±1.55) (Fig. 2). Within the test groups, CHX i.e. the chlorhexidine disinfection group showed the highest SBS (17.34±4.06) which was not statistically significant from CTRL (*P*=.958) ( Table 1). NaOCl (sodium hypochlorite disinfection group) showed the least bond strength (7.60±1.46), which was significantly lower than all other groups (*P*<0.05). The SBS value of the PAD (12.29±2.90), where photo- activated disinfection was used, showed a significantly higher SBS than NaOCl (*P*= .002), but was significantly lower than CTRL and CHX (*P*<0.05).

**Table 1:**
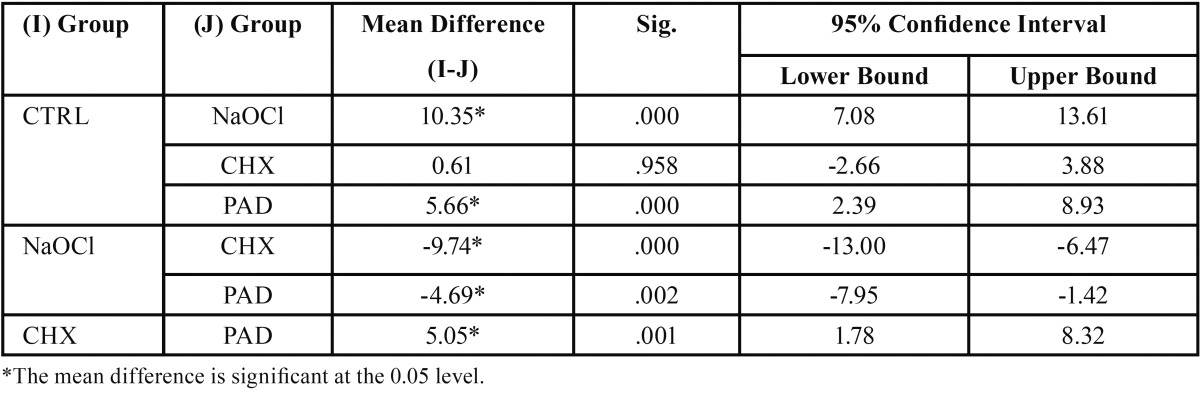
Intergroup comparison.

## Discussion

Clinical scenarios where the disinfection of tooth surface is preferred before the placement of resin modified glass ionomer cement (RMGIC) can be as follows: (a) in operative dentistry, when it is used as a cavity liner or interim restoration or definitive restoration, (b) in endodontics, when it is used for core build-up or perforation repair or intra-orifice sealing. The disinfection of the prepared cavity or the pulp chamber in these situations can minimize the risk for secondary caries or infection via eliminating the remaining microorganisms.(10,11) However, it is important that these disinfectants by themselves have no negative effect on the adhesion of RMGIC.

Glass ionomer cement (GIC) is the only restorative material that truly adheres to the tooth surface. It bonds to the inorganic portion of the tooth through a process referred to as ion exchange reaction (12). Bonding of the carboxyl ion of GIC to the collagen of dentin have also been hypothesized (13,14). Additionally, mechanical interlocking of the cement into dentinal irregularities also have been suggested (15). In RMGIC, the resin component is thought to form a hybrid layer with dentin to aid in adhesion (16). For a good bonding of GIC to the dentin, a clean dentinal surface is a pre-requisite. Hence, any treatment, such as disinfection, that alters the dentinal surface can potentially alter the bond strength of GIC to the dentin. In this study, macro-shear bond-strength (SBS) testing was used to assess the effect of dentin disinfection methods on the bonding of RMGIC as it is an effective, fast and easy method which does not require elaborate specimen preparation (17).

Sodium hypochlorite, a strong oxidizing agent, is a well-known disinfectant. In addition to its antibacterial property, it is an organic solvent (18). In the current study, the use of 1% NaOCl as a cavity disinfectant resulted in the least SBS of RMGIC to the dentin amongst all the groups. This could be attributed to the dissolution of the dentinal collagen fibers due to its proteolytic capability, which can hinder both the chemical bonding of carboxyl ion of GIC to the dentin collagen as well as the formation of hybrid layer (6). Additionally, the oxidizing agent could have also interfered with the polymerization of the resin component of RMGIC (19,20).

Chlorhexidine (CHX), a widely used broad spectrum antimicrobial agent with the property of substantivity is shown to be effective in reducing the cultivable microbiota in contaminated dentin (21). Apart from its antimicrobial property, CHX can improve the durability of the dentin-restorative bond because of its anti-collagenolytic action. This is attributed to the inhibitory effect of CHX on matrix metalloproteinases (22). As per the observations made in this study, it could be stated that 2% CHX was the only cavity cleanser that did not significantly reduce the bond strength of RMGIC to dentin. This was in accordance with the study by Cunningham, *et al.* (3), Aykut-Yetkiner, *et al.* (23), and Ersin, *et al.* (24). The reason for the insignificant reduction in the SBS of RMGIC in Group III could be because of the negligible interference in the setting reaction of GIC by CHX.

Photo-activated disinfection (PAD) is widely used for disinfecting radicular dentin in endodontics (25) and debridement of perio-dontal pockets in periodontics (9). Considering these applications, the use of PAD can be extended to restorative dentistry as well. Lee *et al.* (26) reported that diode laser can eliminate the *Streptococcus mutans* within the residual carious dentin without any pulpal damage when the remaining dentin thickness is greater than 1 mm. In the present study diode laser of 810 nm was used in conjunction with 1% methylene blue dye to disinfect the dentin surface. The use of a chemical mediator, such as methylene blue, in addition to enhancing the antimicrobial action can also serve to act as a heat-sink for the thermal energy (27,28). In this study, the use of 810 nm diode laser after application of 1% methylene blue dye reduced the shear bond strength (SBS) significantly compared to the control group and the CHX group. But the bond strength was significantly higher than the NaOCl group. The decrease in bond strength could be attributed either to the interference of polymerization of the resin component of RMGIC in the presence photo-sensitizing agent or due to the surface change in the dentin caused by the diode laser (29). The result of the current study warrants further investigation to ascertain the effect of diode laser used in different power settings in the presence of other photo-sensitizers on the dentin surface.

## Conclusions

Though the use of disinfectants in restorative dentistry is a controversy, its use in pulp space disinfection is an absolute must. Whatever the clinical scenario, the dentin disinfection protocol followed should be in sync with the restorative material that is to be used. From the results of present *in-vitro* study, it could be concluded that the use of chlorhexidine based cavity disinfectants do not significantly interfere with the adhesion of RMGIC. However, cavity disinfection using 810 nm diode laser and a photo-sensitizer requires further investigations and should be used with prudence. Furthermore, sodium hypochlorite based cavity cleansers should be avoided prior to the use of RMGIC.
